# ﻿New species and records of *Chapsa* (Graphidaceae) in China

**DOI:** 10.3897/mycokeys.85.76040

**Published:** 2021-12-10

**Authors:** Ming-Zhu Dou*, Min Li*, Ze-Feng Jia

**Affiliations:** 1 College of Life Sciences, Liaocheng University, Liaocheng 252059, China Liaocheng University Liaocheng China

**Keywords:** Ascomycota, lichenized fungi, phylogeny, taxonomy

## Abstract

We studied the genus *Chapsa* in China based on morphological characteristics, chemical traits and molecular phylogenetic analysis. One species new to science (*C.murioelongata* M.Z. Dou & M. Li) and two records new to China were found (*C.wolseleyana* Weerakoon, Lumbsch & Lücking and *C.niveocarpa* Mangold). *Chapsamurioelongata***sp. nov.** is characterised by its lobed thalline margin, orange discs with white pruina, clear hymenium, and submuriform and long ascospores. *Chapsawolseleyana* was recombined into *Astrochapsa* based on phenotypic traits. Sequences of this species are for the first time reported here and phylogenetic analyses of three loci (mtSSU, ITS and nuLSU) supported the position of this species within *Chapsa*. A key for the *Chapsa* species known in China is provided.

## ﻿Introduction

The lichen genus *Chapsa* (Graphidaceae) was first established by Massalongo (1860) with *C.indica* as the type species. This genus was ignored for a long time until 2006, when Frisch re-established *Chapsa*, based on the *Chroodiscus*-type apothecia, presence of periphysoids and *Chapsa*-type paraphyses. Frisch (2006) also provided a detailed description and delimitation of the genus *Chapsa*, which was widely recognised by subsequent researchers (Mangold 2008; Frisch and Kalb 2009; Rivas Plata et al. 2011; Sipman et al. 2012; Xu et al. 2016). The genus *Chapsa* was considered to be monophyletic in the beginning (Frisch 2006) but with further research, it was suspected to be polyphyletic (Mangold 2008; Papong et al. 2010). Subsequently, seven genera, *Astrochapsa* Parnmen, Lücking & Lumbsch, *Crutarndina* Parnmen, Lücking & Lumbsch, *Gintarasia* Kraichak, Lücking & Lumbsch, *Pseudochapsa* Parnmen, Lücking & Lumbsch, *Pseudotopeliopsis* Parnmen, Lücking & Lumbsch, *Myriochapsa* M. Cáceres, Lücking & Lumbsch and *Nitidochapsa* Parnmen, Lücking & Lumbsch were separated from *Chapsa*, based on a combination of molecular evidence, phenotypic and chemical characteristics (Parnmen et al. 2012, 2013; Kraichak et al. 2013).

Although China is rich in lichenised fungal species (Wei 2020), there are few studies and reports on the genus *Chapsa*. More than 60 species of *Chapsa* have been reported in the world, of which only three, *C.indica* A. Massal, *C.mirabilis* A. (Zahlbr.) Lücking and *C.leprocarpa* (Nyl.) Frisch, have so far been found in China (Rivas Plata et al. 2010; Xu et al. 2016; Jia and Lücking 2017; Kalb and Kalb 2017; Wijayawardene et al. 2017; de Lima et al. 2019).

During the study of *Chapsa* A. Massal. in southern China, one species, *C.murioelongata* was found new to science, and two species, *C.niveocarpa* Mangold and *C.wolseleyana* Weerakoon, Lumbsch & Lücking were found new to China. In our study, 26 sequences were newly generated from freshly collected specimens.

## ﻿Materials and methods

### ﻿Morphological and chemical analyses

The specimens were collected from southern China and deposited in the Fungarium, College of Life Sciences, Liaocheng University, China (LCUF). Morphological and anatomical characters of thalli and apothecia were examined and photographed under an Olympus SZX16 dissecting microscope and an Olympus BX53 compound microscope. The lichen secondary metabolites were detected and identified by thin-layer chromatography using solvent C (Orange et al. 2010; Jia and Wei 2016).

### ﻿DNA extraction, PCR sequencing and phylogenetic analysis

Genomic DNA was extracted from ascomata using the Hi-DNA-secure Plant Kit (Tiangen, Beijing, China) according to the manufacturer’s protocol. The nuLSU, ITS and mtSSU regions were amplified using the primer pair AL2R/LR6 (Mangold 2008, Vilgalys and Hester 1990), ITS1F/ITS4 (Gardes and Bruns 1993, White et al. 1990) and mrSSU1/mrSSU3R (Zoller et al. 1999), respectively. The PCR amplification progress followed Dou et al. (2018) and the PCR products were sequenced by Biosune Inc. (Shanghai). The newly generated sequences were submitted to GenBank (Table 1).

**Table 1.** Information for the sequences used in this study. Newly generated sequences are shown in bold.

Species	Specimen No.	Locality	ITS	nuLSU	mtSSU
*Pseudochapsaphlyctidioides*	Lumbsch 20500d	Fiji	–	JX465301	JX421005
*Pseudochapsadilatata*	Luecking 32101	Venezuela	–	JX421446	JX420981
*Pseudochapsaesslingeri*	Caceres s.n.	Brazil	–	–	JX420983
*Pseudochapsaesslingeri*	Caceres 6006a	Brazil	–	–	JX420984
*Pseudochapsaesslingeri*	Rivas Plata 107C (F)	Peru	–	–	JX420985
*Pseudochapsaesslingeri*	Rivas Plata 809a (F)	Peru	–	–	JX420986
*Chapsaalborosella*	Luecking 31238a	Brazil	–	JX421439	JX420972
*Chapsaalborosella*	Luecking 25587	Guatemala	–	JX421440	JX420973
*Chapsasoredicarpa*	Luecking 31200	Brazil	–	JX421462	JX421011
*Chapsasoredicarpa*	Luecking 31240	Brazil	–	JX421463	JX421012
*Chapsasublilacina*	Luecking RLD056	Mexico	–	HQ639624	HQ639600
*Chapsathallotrema*	Lucking 32019	Venezuela	–	JX465319	JX421013
*Chapsaindica*	Parnmen018486(RAMK)	Thailand	–	JX465295	JX465280
*Chapsaleprocarpa*	GZ19531	China, Guizhou	**MW009079**	**MW007981**	**MW010276**
*Chapsaleprocarpa*	GZ19537	China, Guizhou	**MW009077**	**MW007984**	**MW010278**
*Chapsaleprocarpa*	GZ19536	China, Guizhou	**MW009080**	**MW007982**	**MW010274**
*Chapsaniveocarpa*	HN19508	China, Hainan	**MW009076**	**MW010272**	–
*Chapsaniveocarpa*	Lumbsch_19125k2(F) & Mangold (F)	Australia, Queensland	–	–	EU675274
*Chapsaniveocarpa*	Lumbsch 19151p & Mangold (F)	Australia, Queensland	–	FJ708487	EU075567
*Chapsapatens*	FJ19131	China, Fujian	**MT995055**	**MW007979**	**MW010275**
*Chapsapatens*	FJ19049	China, Fujian	**MW007918**	**MW007980**	–
*Chapsawolseleyana*	FJ19158	China, Fujian	**MW009078**	**MW010273**	**MW010277**
*Chapsawolseleyana*	FJ19148	China, Fujian	**MW009106**	**MW010270**	**MW010279**
*Chapsamurioelongata*	HN19222	China, Hainan	**MW009102**	**MW010271**	–
*Chapsamurioelongata*	HN19682	China, Hainan	**MW009103**	**MW010269**	–
*Chapsapulchra*	CHAPUL19129t	Australia	–	KC020261	KC020255
*Astrochapsameridensis*	Luecking 17770 (F)	Costa Rica	–	EU075655	EU075610
*Astrochapsamastersonii*	Lumbsch 20500f	Fiji	–	–	JX420996
*Astrochapsazahlbruckneri*	Papong 6516	Thailand	–	JX421467	–
*Astrochapsaastroidea*	Lumbsch 19166n & Mangold(F)	Australia, Queensland	–	EU075614	EU075566
*Astrochapsaastroidea*	Lumbsch 19750a	Thailand	–	JX421441	JX420974
*Astrochapsaastroidea*	Papong 6004	Thailand	–	JX421442	JX420975
*Astrochapsaastroidea*	Luecking 24006	Thailand	–	JX421443	JX420977
*Astrochapsaastroidea*	Luecking 24008	Thailand	–	JX421444	JX420978
*Astrochapsaastroidea*	Luecking 24011	Thailand	–	JX421445	JX465278
*Chroodiscuscoccineus*	Herb. R. Luecking 2000	Costa Rica	–	AF465441	–

Multi-locus (ITS, mtSSU and nuLSU) phylogenetic analysis was performed. The combined analysis included 70 sequences (Table 1) representing 18 in-group taxa and one out-group taxon. As many species as possible of *Chapsa* s. lat. were contained in our data matrix including the taxa that were similar in morphology or sequence to the new species and the two records. We blasted sequences of the three species in GenBank and selected sequence-similar taxa on a pre-determined cut-off.

The alignment was undertaken by applying MAFFT 7 with the option of L-INS-I (Katoh and Standley 2013). The three single-locus alignments were concatenated in PhyloSuite v1.2.2 (Zhang et al. 2020). The concatenated data matrix comprised 3188 nucleotide sites (nuLSU 1405 bp, ITS 647 bp and mtSSU 1136 bp). In order to check the consistency between the three loci, incongruence length difference test (ILD Test) was carried out using PAUP. The P value of ILD Test was 0.65 (>0.5), so the three loci were suitable for polygenic phylogeny. Construction of the ML (Maximum Likelihood) tree was undertaken by applying RAxML v.8.2.12 (Stamatakis 2014) and implementing a GTRGAMMA model. For BI (Bayesian Inference) analysis, PartitionFinder 2 (Lanfear et al. 2017) was used to determine the best-fit model for each partition. For the nuLSU region, we used GTR+I+G, for ITS, GTR+G, and for mtSSU, HKY+I+G. BI analysis was performed with MrBayes 3.2.7 (Ronquist et al 2012). Markov Chain Monte Carlo (MCMC) chains were run for 200,000 generations, sampling every 100^th^ generation, at which point, the average standard deviation of split frequencies was 0.001738. ML bootstrap values (BS) ≥ 75% and Bayesian posterior probabilities (PP) ≥ 0.95 were considered as significantly supported.

## ﻿Results and discussion

**Figure 1.**
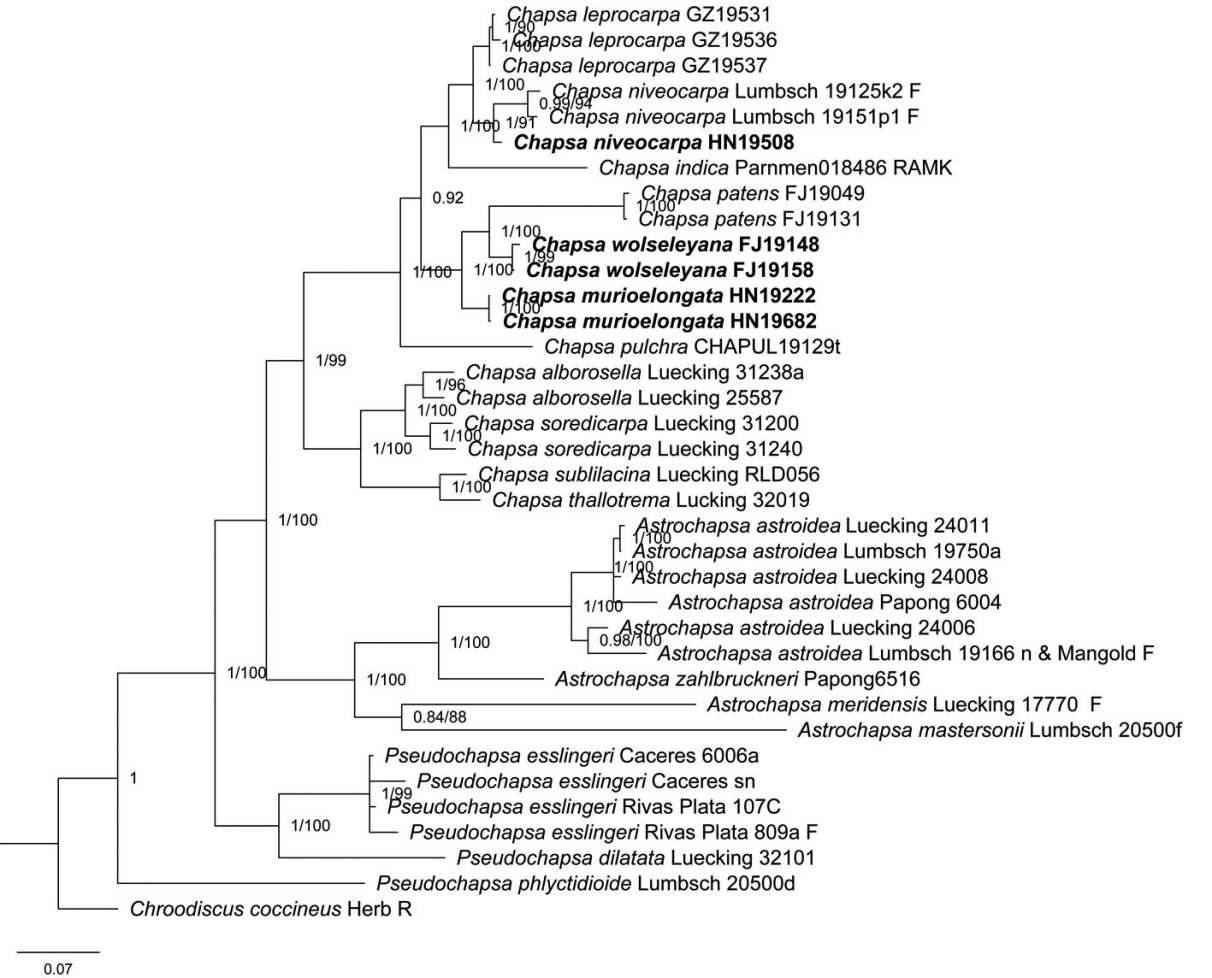
Bayesian phylogenetic tree generated from analysis of combined ITS, nuLSU and mtSSU. *Chroodiscuscoccineus* is the out-group taxon. ML-bootstrap values/Bayesian posterior probabilities above 50% are written next to nodes.

The BI and ML trees showed similar topologies and thus, only the BI tree was provided (Fig. 1). The three species were all monophyletic with a high support value: *C.murioelongata* (100%, 1.00), *C.wolseleyana* (99%, 1.00) and *C.niveocarpa* (91%, 1.00). *Chapsamurioelongata* is sister to the clade consisting of *C.wolseleyana* and *C.patens* (Nyl.) Frisch. *Chapsaniveocarpa*HN19508 and *C.niveocarpa* Lumbsch form a well-supported clade and are sisters to *C.leprocarpa*.

## ﻿Taxonomy

### New species

#### ﻿ Chapsa murielongata

Taxon classificationFungiOstropalesGraphidaceae

M.Z. Dou & M. Li sp. nov.

21828E61-FDA6-5856-94A0-7F50A7BDBDAE

Fungal Names: FN 570754

Figure 2

##### Etymology.

The specific epithet *murioelongata* refers to the elongate, muriform ascospores.

##### Type.

China. Hainan Province: Ledong County, Jianfengling National Forest Park, 18°42'39"N, 108°52'37"E, alt. 760 m, on bark, 09 Dec 2019, Y. H. Ju HN19222 (LCUF: holotype: HN19222; GenBank MW009102 for ITS and MW010271 for LSU).

##### Description.

Thallus corticolous, crustose, olive-grey, surface dull, smooth to uneven, ecorticate. Apothecia erumpent, dispersed or two to four aggregated, rounded, 1–3 mm diam.; thalline margin lobed with white felt-like inner surface, lobes strongly backward curved; disc flesh-coloured, covered by thick, white pruina. Exciple 80–105 μm wide laterally, dark brown; epihymenium 20–40 μm high, with coarse greyish granules; hymenium clear, 110–170 μm high, non-amyloid; hypothecium colourless, 10–30 μm high; paraphyses simple, tips unbranched; periphysoides present, 5–30 μm long. Asci 4–6 (8)-spored, clavate, 100–120 × 35–50 μm; ascospores hyaline, bacillary with rounded to subacute ends, submuriform with 20–25 transverse septa and 0–2 longitudinal septa per segment, 75–105 × 9.5–16 μm, non-halonate, I-. Pycnidia not observed.

**Figure 2.**
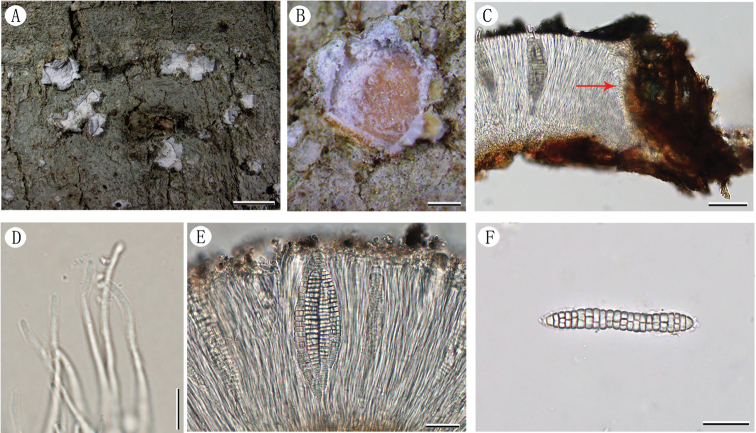
*Chapsamurioelongata* (LCUFHN19222) **A** habit of thallus with apothecia at different developmental stages **B** apothecium (the pruina of the disc partly scraped off) **C** section of apothecium with periphysoids (direction of arrow) **D** paraphyses **E** an ascus containing six ascospores **F** ascospore. Scale bars: 3 mm (**A**); 0.5 mm (**B**); 50 μm (**C**); 8 μm (**D**); 30 μm (**E**); 25 μm (**F**).

##### Chemistry.

Thallus K-, C-, PD-; no compounds detectable by TLC.

##### Ecology and distribution.

On the bark in semi-exposed forest of Hainan Province.

##### Additional specimens examined.

China. Hainan Province: Changjiang County, Bawangling Nature Reserve, Yajia Scenic Area, 10°04'54"N, 109°07'04"E, alt. 810 m, on bark, 08 Dec 2019, Y. H. Ju HN19167 (LCUF); China. Hainan Province: Lingshui County, Diaoluo Mountain, 18°43'35"N, 109°52'02"E, alt. 900 m, on bark, 14 Dec 2019, M. Li HN19682 (LCUF) (GenBank MW009103 for ITS and MW010269 for LSU).

##### Note.

*Chapsamurioelongata* is characterised by its olive-grey thallus; white pruinose discs; distinct periphysoids; clear hymenium; 4–8-spored asci; submuriform ascospores with 20–25 transverse septa and 0–2 longitudinal septa per segment. *Chapsamicrospora* Kalb, *C.asteliae* (Kantvilas & Vězda) Mangold, *Astrochapsaelongata* Poengs. & Lumbsch and *C.patens* are morphologically similar to the new species. *Chapsamicrospora* can be distinguished from *C.murioelongata* by the smaller apothecia (0.6–1.2 mm diam.), transversely septate and smaller ascospores (7–9 × 4 μm) (Lumbsch et al. 2011). *Chapsaasteliae* differs in amyloid and shorter ascospores (30–80 μm) (Kantvilas and Vězda 2000; Mangold 2008). *Astrochapsaelongata* differs from *C.murioelongata* in having shorter ascospores (40–65 μm) and less longitudinal septa per segment (0–1) (Poengsungnoen et al. 2019). *Chapsapatens* differs from *C.murioelongata* chiefly in the single-spored asci and broader ascospores (22–35 μm) (Frisch 2006).

Blast searches of nuLSU sequences indicate *Chapsamurioelongata* has close affinities with *C.patens* (98.36% identity), *C.wolseleyana* (95.63% identity), *C.leprocarpa* (91.97% identity) and *C.indica* (90.81% identity), so all these species were included in the phylogenetic analyses. *Chapsamurioelongata* was well separated from any other species in the tree and strongly supported as the monophyletic (PP = 1; ML = 100%).

### New records

#### ﻿ Chapsa wolseleyana

Taxon classificationFungiOstropalesGraphidaceae

Weerakoon, Lumbsch & Lücking, in Weerakoon, Rivas Plata, Lumbsch & Lücking, Lichenologist 44(3): 377 (2012)

A4D52B9B-0B9F-562A-98A4-9C5C6DB8B6F7

Figure 3

Astrochapsa wolseleyana (Weerakoon, Lumbsch & Lücking) Parnmen, Lücking & Lumbsch, in Parnmen et al., PLoS ONE 7(12): 10 (2012)

##### Description.

Thallus crustose, corticolous, grey-brown, surface dull to slightly shiny, uneven, fissured. Apothecia erumpent, dispersed, sometimes two or three fused, mostly rounded to seldom slightly angular, 0.7–1.2 mm diam.; thalline margin raised to lobulate, lobes erected to recurved, inner part brown, covered with rose-red or white pruina; disc exposed, rose-red, covered with thick, rose-red pruina. Exciple fused, cupular, laterally 180–250 μm wide, yellowish-brown to brown; epihymenium rose-red with granules, 20–50 μm high, K+ green; hymenium 140–230 μm high, clear, colourless, non-amyloid; hypothecium indistinct; paraphyses septate, tips rose-red and moniliform with oval or rectangular cells; periphysoides present, 50–100 μm long. Asci clavate, 1-spored, 110–135 × 35–50 μm; ascospores densely muriform, oblong-ellipsoid, with hemispherical to roundish ends, 105–130 × 30–45 μm, first reddish, becoming hyaline to slightly olive-brown at maturity, I-. Pycnidia not observed.

**Figure 3.**
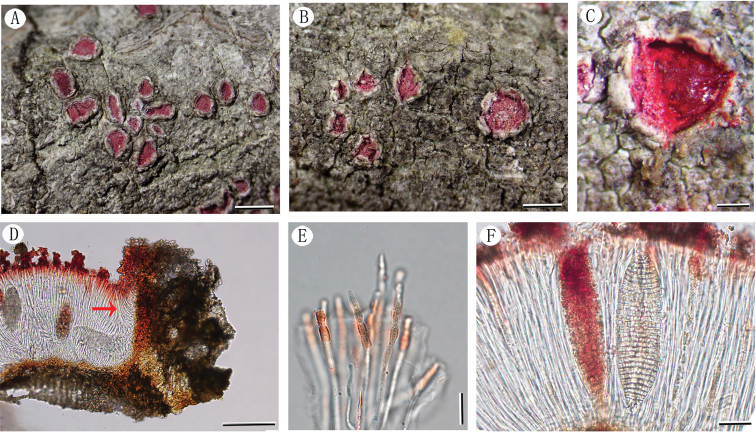
*Chapsawolseleyana* (LCUF FJ19148-b) **A** habit of thallus with apothecia **B** apothecia at different developmental stages **C** apothecium (part of pruina scraped off) **D** section of apothecium with periphysoids (direction of arrow) **E** paraphyses **F** young and mature ascospores. Scale bars: 1.5 mm (**A**); 1 mm (**B**); 0.25 mm (**C**); 120 μm (**D**); 10 μm (**E**); 25 μm (**F**).

##### Chemistry.

No substances detected by TLC but apothecial disc with pigment producing K+ yellow-green efflux, suggesting presence of isohypocrelline.

##### Ecology and distribution.

Growing on bark exposed to wind and high light intensity in montane forests. Worldwide distribution: Sri Lanka (Weerakoon et al. 2012) and newly reported for China.

##### Selected specimens examined.

China. Fujian Province: Quanzhou City, Jiuxian Mountain, Reflecting Pool, 25°42'57"N, 118°07'14"E, alt. 1540 m, on bark, 5 Jul 2019, F.Y. Liu FJ19148-b (LCUF) (GenBank MW009106 for ITS, MW010270 for LSU and MW010279 for SSU); China. Fujian Province: Quanzhou City, Jiuxian Mountain, Natural Observation Path, 25°42'44"N, 118°07'17"E, alt. 1460 m, on bark, 25 Jul 2019, F.Y. Liu FJ19158 (LCUF) (GenBank MW009078 for ITS, MW010273 for LSU and MW010277 for SSU). China. Fujian Province: Quanzhou City, Jiuxian Mountain, Reflecting Pool, 25°42'57"N, 118°07'14"E, alt. 1540 m, on bark, 25 Jul 2019, F.Y. Liu FJ19127-2, same locality, FJ19128-2, FJ19141-2 (LCUF).

##### Note.

*Chapsawolseleyana* is characterised by its grey-brown, uneven thallus, apothecia with raised to lobed thalline margin, rose-red discs with similar coloured pruina, rose-red epihymenium and paraphyses tips, distinct periphysoids, 1-spored asci, muriform ascospores, red when young and hyaline to olive-brown when old. Only a few species of *Chapsa* have pigmented discs and among them *C.rubropulveracea* Hale ex Mangold, Lücking & Lumbsch is morphologically most similar to *C.wolseleyana*, but its thallus is farinose and its ascospores are 8 per ascus, smaller (15–20 ×5–6 μm) and transversely septate (Lumbsch et al. 2011).

*Chapsawolseleyana* was transferred to *Astrochapsa*, based on a phenotype-based analysis (not molecular phylogeny) (Parnmen et al. 2012). However, our phylogenetic analysis shows that this species belongs in *Chapsa*, rather than *Astrochapsa. Chapsawolseleyana* was associated phylogenetically with a strongly-supported clade (100/1) with *C.patens*, but with sufficient distance to be considered a distinct species. In addition, the latter differs from *C.wolseleyana* in having larger pale brown apothecia (up to 2 mm diam.) with white pruina, unpigmented epihymenium and unpigmented paraphyses adspersed with fine greyish to brownish granules, hyaline ascospores (Frisch et al. 2006; Joshi et al. 2012; Joshi et al. 2018).

#### ﻿ Chapsa niveocarpa

Taxon classificationFungiOstropalesGraphidaceae

Mangold in Mangold, Elix & Lumbsch, Flora of Australia, 57:654 (2009)

2503DFE0-D91C-5C13-8932-F4933E7B7633

Figure 4

##### Description.

Thallus corticolous, crustose, pale grayish-green surface dull and fluctuating along the bark. Apothecia erumpent, solitary to fused, angular rounded to slightly elongate, 0.5–1.8 × 0.5–1.2 mm; thalline margin split and recurved, insidewith thick white pruina; disc exposed, yellowish-brown, covered by white pruina. Exciple laterally 12–75 μm wide, dark brown; epihymenium 10–20 μm high; hymenium 120–200 μm high, grey-brown, inspersed by granules, non-amyloid; hypothecium indistinct; paraphyses unbranched; tips distinctly thickened; periphysoides present, but obscured by granular inclusions. Asci 1-spored, clavate, 120–140 × 27–36 μm; ascospores densely muriform, with thick halo at both ends, oblong, hyaline, 115–135 × 25–34 μm, I-. Pycnidia not observed.

**Figure 4.**
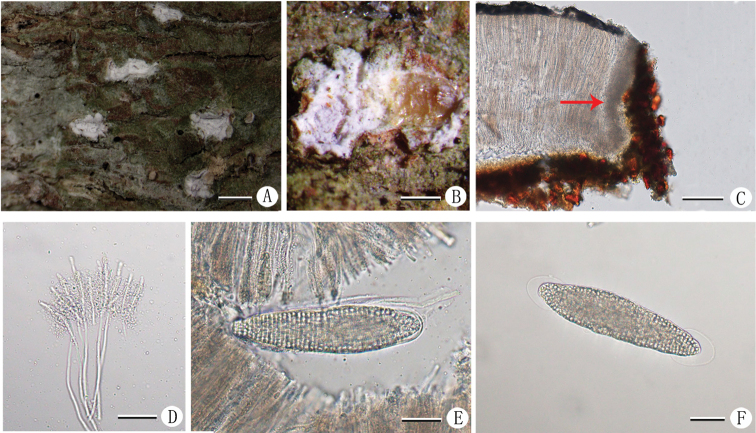
*Chapsaniveocarpa* (LCUFHN19508) **A** habit of thallus with apothecia **B** apothecium (part of pruina scraped off) **C** section of apothecium with periphysoids (direction of arrow) **D** paraphyses with hyaline granules **E** ascus **F** ascospore with halo. Scale bars: 1 mm (**A**); 0.5 mm (**B**); 50 μm (**C**); 25 μm (**D**); 30 μm (**E**); 25 μm (**F**).

##### Chemistry.

Thallus K-, C-, PD-; no compounds detectable by TLC.

##### Ecology and distribution.

Growing on tree bark in tropical rainforests in altitudes ranging from 500 to 1100 m. Australia, Queensland (Mangold 2008); newly reported for China.

##### Selected specimens examined.

China. Hainan Province: Wuzhishan City, Wuzhishan Nature Reserve, 18°54'13"N, 109°41'04"E, alt. 870 m, on bark, 12 Dec 2019, M. Li HN19508 (LCUF) (GenBank MW009076 for ITS and MW010272 for LSU); China. Hainan Province: Wuzhishan City, Wuzhishan Nature Reserve, 18°53'13"N, 109°41'04"E, alt. 1020 m, on bark, 12 Dec 2019, M. Li HN19530 (LCUF); China. Hainan Province: Wuzhishan City, Wuzhishan Nature Reserve, 18°54'13'N, 109°41'04'E, alt. 870 m, on bark, 12 Dec 2019, M. Li HN19499 (LCUF); China. Hainan Province: Lingshui County, Diaoluo Mountain, 18°43'35"N, 109°52'02"E, alt. 900 m, on bark, 14 Dec 2019, M. Li HN19687 (LCUF); China. Hainan Province: Lingshui County, Diaoluo Mountain, 18°43'35"N, 109°52'02"E, alt. 900 m, on bark, 14 Dec 2019, M. Li HN19679 (LCUF).

##### Note.

*Chapsaniveocarpa* is characterised by its crustose, pale greyish-green thallus; rounded to elongate apothecia, yellowish-brown discs with white pruina, obscured periphysoids, inspersed hymenium, 1-spored(rare 2-spored)ascus and muriform and hyalineascospores with halo. *Chapsaniveocarpa* is morphologically similar and phylogenetically related to *C.leprocarpa*, and both species occur on bark in tropical forests (Frisch 2006; Mangold 2008; Parnmen et al. 2012). *Chapsaleprocarpa* differs from *C.niveocarpa* in having a lower hymenium (100–130 μm) and smaller ascospores (up to 111 μm long) (Frisch 2006). The specimen (HN19508) we collected in China is allocated phylogenetically to a strongly-supported (1/91) clade with *C.niveocarpa*. The collections cited above are the first reports for China.

#### ﻿Key to *Chapsa* in China

**Table:** 

1	Disc with red pruina; ascospores 1/ascus, muriform, 105–135 × 30–50 μm	***C.wolseleyana***
–	Disc with white pruina	**2**
2	Ascospores transversely septate; ascospores 4–8/ascus, 50–110 × 6–12 µm	***C.indica***
–	Ascospores (sub)muriform	**3**
3	Hamathecium inspersed; ascospores 1/ascus, 80–190 × 20–50 μm	***4***
–	Hamathecium clear	**5**
4	Ascospores 1/ascus, 80–190 × 20–50 μm	***C.niveocarpa***
–	Ascospores 8/ascus, 40–50 × 11–15 μm	***C.mirabilis***
5	Asci 4‒6 (8)-spored; acsospores oblong to cylindrical with rounded to subacute ends, submuriform with 20–25 transverse septa and 0–2 longitudinal septa per segment, 75–105 × 9.5‒16 μm	***C.murioelongata***
–	Asci 4-spored; acsospores oblong to slightly ellipsoid, with roundish ends, 60–130 × 20–40 μm	***C.leprocarpa***

## Supplementary Material

XML Treatment for Chapsa murielongata

XML Treatment for Chapsa wolseleyana

XML Treatment for Chapsa niveocarpa
